# Characterization of the watercress (*Nasturtium officinale* R. Br.; Brassicaceae) transcriptome using RNASeq and identification of candidate genes for important phytonutrient traits linked to human health

**DOI:** 10.1186/s12864-016-2704-4

**Published:** 2016-05-20

**Authors:** Nikol Voutsina, Adrienne C. Payne, Robert D. Hancock, Graham J. J. Clarkson, Steve D. Rothwell, Mark A. Chapman, Gail Taylor

**Affiliations:** Centre for Biological Sciences, University of Southampton, Southampton, SO17 1BJ UK; Cell and Molecular Sciences, The James Hutton Institute, Dundee, DD2 5DA UK; Vitacress Salads Ltd, Lower Link Farm, St Mary Bourne, Andover SP11 6DB UK

**Keywords:** Watercress, *Nasturtium officinale*, Brassicaceae, RNASeq, *de novo* assembly, Differential expression, Antioxidant capacity, Glucosinolates, Gluconasturtiin, Phenylpropanoid pathway

## Abstract

**Background:**

Consuming watercress is thought to provide health benefits as a consequence of its phytonutrient composition. However, for watercress there are currently limited genetic resources underpinning breeding efforts for either yield or phytonutritional traits. In this paper, we use RNASeq data from twelve watercress accessions to characterize the transcriptome, perform candidate gene mining and conduct differential expression analysis for two key phytonutritional traits: antioxidant (AO) capacity and glucosinolate (GLS) content.

**Results:**

The watercress transcriptome was assembled to 80,800 transcripts (48,732 unigenes); 71 % of which were annotated based on orthology to *Arabidopsis*. Differential expression analysis comparing watercress accessions with ‘high’ and ‘low’ AO and GLS resulted in 145 and 94 differentially expressed loci for AO capacity and GLS respectively. Differentially expressed loci between high and low AO watercress were significantly enriched for genes involved in plant defence and response to stimuli, in line with the observation that AO are involved in plant stress-response. Differential expression between the high and low GLS watercress identified links to GLS regulation and also novel transcripts warranting further investigation. Additionally, we successfully identified watercress orthologs for *Arabidopsis* phenylpropanoid, GLS and shikimate biosynthesis pathway genes, and compiled a catalogue of polymorphic markers for future applications.

**Conclusions:**

Our work describes the first transcriptome of watercress and establishes the foundation for further molecular study by providing valuable resources, including sequence data, annotated transcripts, candidate genes and markers.

**Electronic supplementary material:**

The online version of this article (doi:10.1186/s12864-016-2704-4) contains supplementary material, which is available to authorized users.

## Background

Watercress, *Nasturtium officinale* R. Br. (Brassicaceae), is a perennial dicotyledonous herb usually found in close proximity to water [1]. As a member of the Brassicaceae, it is related to several popular food and spice crops, such as broccoli, cabbage, kale, radish and mustard, as well as the model plant *Arabidopsis thaliana* (L.) Heynh. The consumption of Brassicaceae vegetables is suggested to benefit human health as a consequence of their phytochemical composition, which includes high concentrations of glucosinolates (GSL) [2–4]. In particular, watercress has been used as a medicinal and food crop for over 2000 years [5]. Over the past few decades, a growing number of studies suggest that watercress consumption supports health by providing chemopreventive, antioxidant and anti-inflammatory benefits. Specifically, several studies have shown that watercress extracts can act in vitro to combat the growth and metastasis of cancer cells [6–10]. The consumption of watercress by adults also limited exercise-induced DNA damage [11] and increased blood antioxidants [12, 13]. Recently, it was ranked as the top “powerhouse fruit and vegetable” with the strongest link to decreased occurrence of chronic disease [14], ranking highly because it contains an array of both essential nutrients as well as non-essential health-promoting phytochemicals.

Two pivotal traits contributing to the watercress phytonutritient profile are antioxidant (AO) capacity and GLS content. As plant-derived AOs are thought to be an important source of health benefits associated with vegetable and fruit consumption [15], maintaining or increasing AO capacity of food crops is the principal aim of several research and breeding programs [16–19]. Several types of dietary AOs are derived from the phenylpropanoid pathway, such as phenolic acids and flavonoids [20] and this pathway has been well described in *Arabidopsis* [21]. Three studies have recently described phenolic compounds present in watercress. Santos et al. [22] observed that the major phenolic group in watercress are the flavonols, primarily quercetin, kaempferol and isorhamnetin species. A second study, on baby-leaf watercress, identified chlorogenic acid, quercetin-3-O-rutinoside, caffeoyltartaric acid and isorhamnetin as the most abundant phenolic components [23]. Finally, Martínez-Sánchez et al. [24] demonstrated that watercress leaves contain almost double the amount of polyphenols found in other leafy Brassicaceae crops, namely mizuna, rocket and wild rocket.

GLS, which are secondary plant metabolites with anti- herbivory properties [25], are thought to be responsible for the health benefits and characteristic strong mustard flavour associated with several Brassicaceaes [3, 26]. Upon injury of the plant tissue, GLS are hydrolysed by the enzyme myrosinase to nitriles, thiocyanates and isothiocyanates, the quantities of each dependent on reaction conditions [27, 28]. Isothiocyanates have been studied extensively and are thought to have chemopreventive properties [4, 26]. In addition, evidence suggests that the use of these compounds in association with chemotherapy drugs could increase their effectiveness [29]. Thus, the GLS phenotype is an integral part of the nutritional profile in watercress, as well as contributing to the potent peppery flavour of the crop.

Despite its unique nutritional profile and its global market as a food crop, there is no watercress breeding programme and no genetic and genomic resources are available. Research to date has focused primarily on the biomedical implications of watercress consumption and little is known about the watercress crop as a source of germplasm for breeding and improvement. Particularly limited are the genetic resources available to inform industry and science in future improvement or preservation of these important nutritional traits in the crop. To date, selection for important agronomic traits, such as frost or disease resistance, has been conducted on a small scale by growers in-house and there no varieties specifically bred for commercial production [30, 31]. In fact, little genetic variation appears to exist amongst commercial watercress [32]. Recently, Payne et al. [33] surveyed differences in morphology of above-ground characteristics in 25 accessions of watercress from the University of Southampton germplasm collection, which maintains germplasm from growers around the world. The research identified promising range in agronomic characters but limited accession specificity and suggested that breeding could lead to great improvements through selection and the development of varieties. High precision molecular breeding tools could make significant contributions to this crop, especially for the preservation and improvement of traits associated with the high nutritional profile and unique flavour of this crop in future breeding.

Next Generation Sequencing (NGS) technologies provide an opportunity for accelerated crop breeding, even for crops that are considered ‘specialist’ and for which there is no genetic and genomic underpinning knowledge [34]. RNA Sequencing (RNASeq), also known as Whole Transcriptome Shotgun Sequencing, is a method developed to generate a snap-shot of the expressed genome and expression levels within a tissue under a particular set of conditions [35]. This tool can be applied to reveal differences in gene expression under varying environmental conditions, developmental stages, or between phenotypes.

In this study, we present the development of a set of genomic tools for watercress breeding. Specifically, the watercress transcriptome was sequenced using NGS-based Illumina paired-end reads and assembled using the software, Trinity. An annotated catalogue of watercress transcripts was created and differential expression (DE) analysis completed to investigate the genetic basis of two key watercress nutritional attributes: AO capacity and GLS content. Candidate gene mining was also conducted to identify watercress orthologs of known genes in the phenylpropanoid and GLS biosynthetic pathways, and a catalogue of polymorphic markers assembled.

## Results

### Sequencing and de novo assembly

Watercress accessions from the University of Southampton germplasm collection were grown under standard commercial conditions in the U.K, as described previously [33]. Tissue samples were collected at the time of commercial harvest and evaluated for antioxidant (AO) capacity and glucosinolate (GLS) content (Table 1). RNA was extracted from the highest and lowest five samples, as well as two controls of commercial significance. The resulting 12 watercress accessions were sequenced on an Illumina Hiseq2500 generating a total of 323,827,923 paired-end fragments, thus producing an average of 26,985,660 reads per sample: documented in detail in Table 2. Reads have been deposited in the National Center for Biotechnology Information Sequence Read Archive (www.ncbi.nlm.nih.gov/sra) under SRA accession number SRP058520 and BioProject: PRJNA284126. For the commercial watercress accession chosen for the reference assembly (NAS080), 28,128,352 paired-end reads were sequenced. Following quality check and normalization of data, the initial transcriptome was *de novo* assembled using Trinity [36] and contained 87,844 transcripts, which correspond to 48,732 components or “unigenes” (further statistics in Table 3). These numbers did not change greatly when Trinity assembly settings were altered to allow reads with more single nucleotide polymorphisms (SNPs) to be assembled together (See Table 3). A reduction in the allowed gap, to 10 bases between sequences of the same transcript, increased the number of transcripts by 3258 (i.e. there are 3258 transcripts which are merged when a 15 base gap is allowed). The permission of single copy k-mers increased the number of transcripts by 31,672 and genes by 30,469 however these will be enriched for those with little support.

**Table 1:** Phenotypic data describing the watercress sequenced in this project. Antioxidant (AO) capacity was assessed using the FRAP antioxidant assay. Gluconasturtiin concentration, the primary glucosinolate (GLS) in watercress, was assessed using HPLC-MS. Concentration of gluconasturtiin was then quantified for this study as the ratio of the compounds peak area over the peak area of the internal standard (sinigrin). NA specifies that no data or classification is available for that sample

Sample	Antioxidant capacity^a^	AO group	Gluconasturtiin	GLS group
	(mmol Fe2+ equivalent/g fresh weight)		(Peak area ratio)	
NAS080	501	Control	11.1	Control
NAS081	837	High	7.4	Low
NAS057	808	High	15.9	High
NAS092	803	High	12.9	NA
NAS095	841	High	12.0	NA
NAS058	903	High	15.1	High
NAS061	373	Low	14.5	High
NAS068	185	Low	9.4	Low
NAS066	405	Low	11.4	NA
NAS070	271	Low	11.0	NA
NAS093	327	Low	7.0	Low
NAS065	NA	NA	NA	NA

^a^Antioxidant data modified from Payne et al. [33]

**Table 2:** Per sample returns from RNA sequencing on an Illumina Hiseq2500 of 12 samples. This table indicates the total number of fragments sequenced per sample, the number of reads remaining after removal of poor quality reads (Q <15), and the percentage of total reads removed

Sample	Total fragments sequenced	Reads with Q >15	% Reads removed
NAS080	28128352	27988115	0.5
NAS081	25972028	25863143	0.4
NAS057	24014626	23897299	0.5
NAS092	23467409	23383732	0.4
NAS095	24974526	24847043	0.5
NAS058	28504130	28299106	0.7
NAS061	27430238	27263143	0.6
NAS065	30038254	29802658	0.8
NAS068	30021260	29843121	0.6
NAS066	26151350	25992461	0.6
NAS070	28834483	28627104	0.7
NAS093	26291267	26205166	0.3

**Table 3:** Descriptors of the assemblies completed using differing settings to assess the nature of the data. The assemblies use RNASeq data from a commercially active watercress accession, NAS080. Underlined assembly k2g15d2 (k-mer overlap: 2, maximum gap permitted within path: 15 bases, maximum differences allowed within a path: 2) was taken forward as the reference transcriptome for watercress

Assembly	Min k-mer coverage	Max gap allowed	Max differences allowed	Total transcripts	Total components	% GC	N50
k2g10d2	2	10	2	91102	48635	41.12	1587
k2g15d0	2	15	0	87823	48709	41.09	1574
k2g15d2	2	15	2	87844	48732	41.08	1571
k2g15d4	2	15	4	87945	48717	41.09	1574
k2g15d8	2	15	8	87923	48701	41.08	1575
k1g15d2	1	15	2	119516	79201	40.72	1534
k1g15d4	1	15	4	119564	79225	40.74	1532

**Fig. 1:**
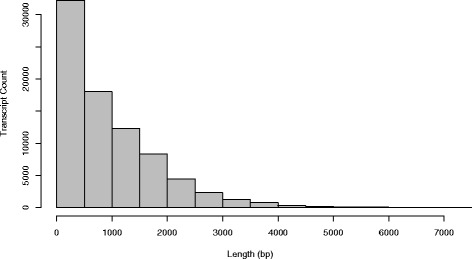
Assembled transcript length distribution. Frequency histogram showing the distribution of transcript length in the watercress reference transcriptome

The selected assembly (k2g15d2; Table 3) was then trimmed to further remove transcripts with low support, reducing the total transcript number to 80,800 (8 % of total transcripts trimmed). The distribution of transcript lengths is shown in Fig. 1. The reference individual’s original reads were mapped back to the reference transcriptome, resulting in successful alignment of 68.9 % of reads (19,294,839 of 27,988,115 reads). Alignment success was consistent across samples sequenced and ranged from 61.4 to 69.6 %, with a mean of 67.4 %. The assembled transcriptome has been submitted to DDBJ/EMBL/GenBank under accession number GEMC00000000.

### Annotation of the watercress transcriptome

**Fig. 2:**
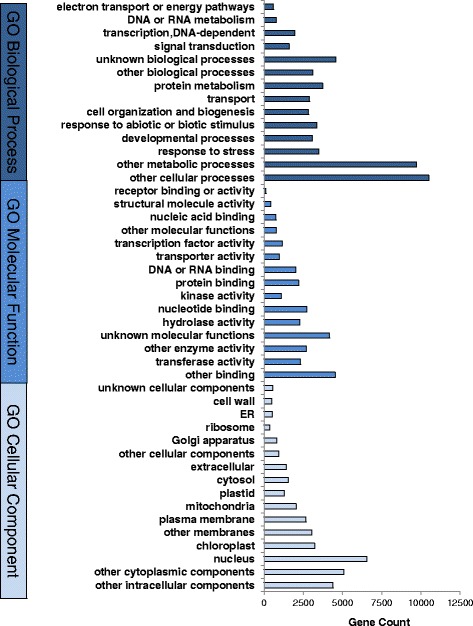
Gene ontology description of the watercress transcriptome. Histogram illustrating the number of genes in the reference watercress transcriptome belonging to GO terms for Biological Process, Molecular Function or Cellular Component categories

Of the 80,800 watercress transcripts, 54,595 (67.6 %) were annotated using a BLASTx search against *Arabidopsis* directly (Additional file 1: Table S1), and mean hit match of watercress to *Arabidopsis* sequences was 84.9 %. An additional 3 % of transcripts were annotated from the UniProtKB/SWISS-PROT database, a further 2480 hits in *Arabidopsis* and 274 hits in other plant species. Throughout the whole transcriptome, the most represented Gene Ontology (GO) categories were ‘other cellular processes’, ‘other binding’, and ‘nucleus’ under each the GO categories biological process, molecular function and cellular component, respectively (Fig. 2). A check for non-nuclear DNA contamination revealed 179 transcripts to be at least 95 % similar to mitochondrial or chloroplast DNA, which were flagged as such (0.2 % of all transcripts).

### Identification of candidate genes

Known *Arabidopsis* AO and GLS biosynthesis pathway gene sequences were queried against the watercress transcriptome using BLASTn. The sequences for several *Arabidopsis* phenylpropanoid pathway enzymes had orthologs; specifically 19 of 24 phenylpropanoid genes queried had at least one close match in the watercress transcriptome (25 transcripts in total). Of the 19 hits, 14 were true orthologs as confirmed by a reciprocal best match BLAST query. The watercress transcripts were an 80.2–94.3 % (mean = 89.0 %) match to the *Arabidopsis* gene sequences.

For the GLS biosynthesis gene queried, 54 of 54 gene sequences were successfully matched to at least one watercress transcript (63 transcripts in total). For these 54 genes, the top hit was further confirmed as an ortholog by reciprocal BLAST query. These transcripts ranged from 81.0 to 94.2 % (mean = 88.5 %) match to the queried *Arabidopsis* sequences. Four *Arabidopsis* loci identifiers (*AT1G62570*, *AT1G62540*, *AT1G62560*, and *AT1G65860*), all corresponding to sequences for the enzyme glucosinolate S-oxygenase, hit the same transcript in this search. In addition, three annotated transcripts (belonging to the same unigene) were identified as a match for the coding sequence of the enzyme myrosinase and three additional transcripts (two unigenes) are described as coding for myrosinase-like proteins.

### Genetic relatedness and polymorphic marker development

There were 46,078 (57.0 % of total transcripts produced) loci with at least 100 bases of sequence without missing data present in all twelve accessions, and these were compared using the software ProSeq3 [37] for the presence of polymorphisms. The number of transcripts containing at least one SNP was 10,134 (22 % of 46,078 transcripts) and 2129 (4.6 %) contained five or more SNPs. Nucleotide diversity indices π [38] and θ [39, 40] were calculated across the dataset, and excluding sites with missing data, the mean π was 0. 78 and the mean θ was 0.87 per kilobase (Kb). In the reference transcriptome, 4972 loci contained at least one Simple Sequence Repeat (SSR) with a total of 5277 SSRs identified. Of these, 54 were compound (two SSRs within 50 bases of each other), 2250 were dinucleotide repeat SSRs, 2448 were trinucleotide repeat SSRs, and 525 were tetranucleiotide repeat SSRs. Seven thousand SNPs were used to draw the phylogenetic relationship between the accessions and is presented in Additional file 2: Figure S1.

### Differential expression between high and low antioxidant watercress

**Fig. 3:**
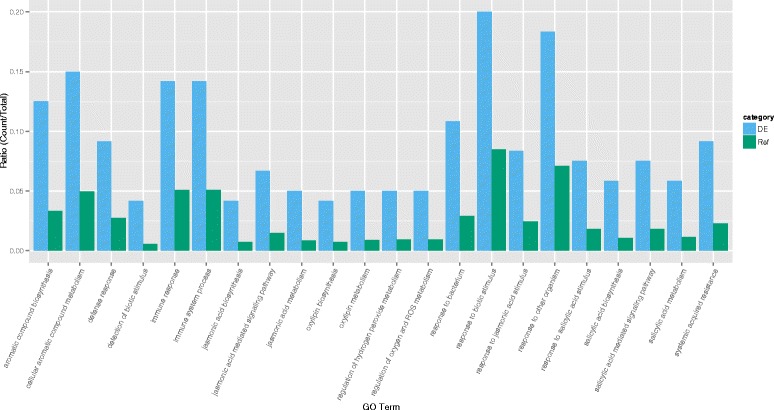
Highlighted gene ontology categories in high antioxidant watercress. Barplot depicting standardized gene count (ratio of gene count in that category over total gene count) of significantly overrepresented GO terms in the AO DE genes in comparison the reference transcriptome

Differential expression analysis was conducted to compare gene expression between five high and low AO watercress previously identified using edgeR [41]. For the AO trait, 145 transcripts (corresponding to 134 genes) were DE at a significance level of FDR ≤ 0.05 (60 transcripts at FDR ≤ 0.01, *n* = 10) (See Additional file 1: Table S1). Fourteen transcripts did not have a BLAST hit and remain of unknown function. Many of the annotated DE loci are associated with mechanisms of stress tolerance, wounding, or response to threat and external stimulus (Additional file 1: Table S1). The AgriGO pipeline confirmed this by highlighting 23 significantly over-represented GO categories in the DE loci, related to immune system response, response to biotic stimulus and stress response functions (See Fig. 3). The raw abundance estimate data for each locus and sample is available in Additional file 3: Table S2.

### Differential expression between high and low glucosinolate watercress

**Fig. 4:**
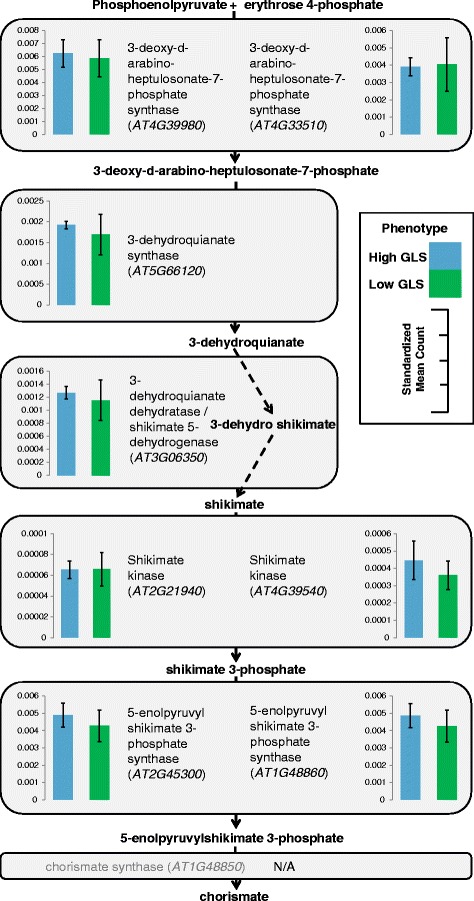
Expression levels throughout the shikimate pathway in high and low GLS watercress. Representation of the shikimate biosynthesis pathway with expression levels, as standardized mean counts (± standard error of the mean), of the best match transcript for high and low glucosinolate accessions. Chorismate synthase (*AT1G48850*) did not have a BLAST hit to the watercress transcriptome

**Fig. 5:**
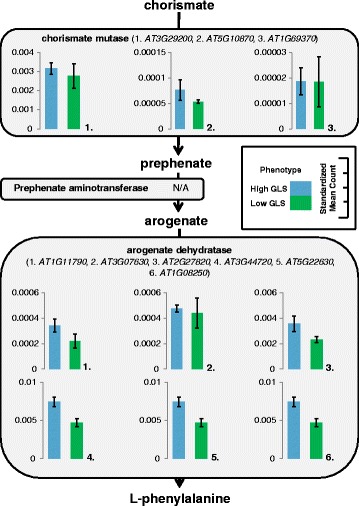
Expression levels throughout phenylalanine biosynthesis in high and low GLS watercress. Representation of the most common phenylalanine biosynthesis pathway in plants with expression levels, as standardized mean counts (± standard error of the mean), of the best match transcript for high and low glucosinolate accessions. Prephenate aminotransferase did not have an available consensus sequence at this time

**Fig. 6:**
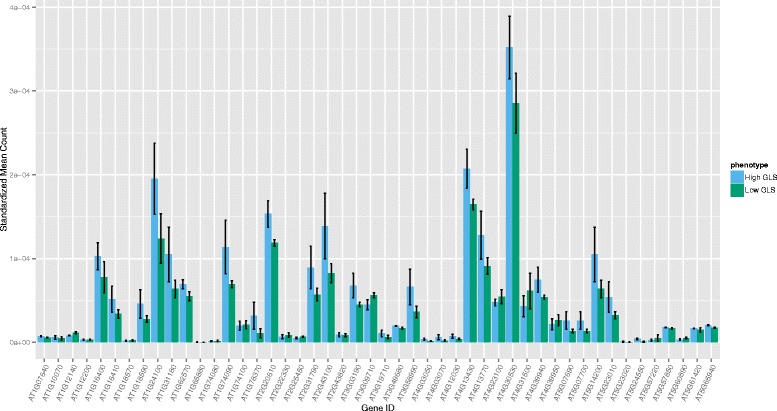
Expression levels of genes in GLS biosynthesis in high and low GLS watercress. Mean expression levels (± standard error of the mean), as standardized mean counts, of watercress transcripts similar to known glucosinolate biosynthesis genes in high and low glucosinolate accessions

The DE analysis for GLS content yielded 94 DE loci at a significance level of FDR ≤ 0.05, corresponding to 93 different genes (50 transcripts at FDR ≤ 0.01, *n* = 6) (See Additional file 1: Table S1). Twenty four of these transcripts did not have a BLAST hit. The functional classification of the 70 annotated loci was completed using AgriGO and yielded only one significant GO term: exopeptidase activity. The DE results revealed several genes with putative functions related to GLS biosynthesis; including 13 stress response genes and two genes associated with the shikimate pathway (*AT3G06350 & AT2G35500*). This pathway results in the production of chorismate which is then converted to phenylalanine [42], the precursor to aromatic GLS, including the most abundant GLS in watercress: gluconasturtiin [43–45]. The shikimate pathway produces chorismate through seven steps involving six enzymes, chorismate is then converted to L-phenylalanine primarily via an arogenate intermediate [42]. We used BLASTn to identify equivalent transcripts to the genes in these pathways, the *Arabidopsis* sequences of which were obtained from the NCBI database. This resulted in discovery of 112 transcripts which matched the 18 known shikimate and phenylalanine biosynthesis pathway gene sequences. The total of standardised expression counts of all transcript isoforms for the best match watercress unigene (lowest e-value and highest score) are shown in Figs. 4 and 5. We also compared expression levels (standardized mean count) of the GLS biosynthesis candidate genes identified previously (Fig. 6). Although the expression of these transcripts was not significantly different between high and low GLS concentration watercress based on the transcriptome-wide analysis (ca. 80,000 loci), there was a noticeable trend of up-regulation of genes involved in the shikimate (15/17) and GLS (39/54) biosynthetic pathways in the high GLS watercress. The raw abundance estimate data for each locus and sample is available in Additional file 4: Table S3.

### Sequence divergence between high and low accessions

Strong sequence divergence between high and low AO and GLS accessions would be expected for loci involved in these pathways. We therefore calculated F_ST_ for all loci in ProSeq3. At a cut-off of F_ST_ = 0.5, 608 (of 19,229) and 306 (of 21,924) loci showed high sequence divergence between high/low AO and high/low GLS groups, respectively. Some of the loci in this subset may play a role in governing AO or GLS biosynthesis.

This comparison between high/low AO groups revealed only three loci with fixed differences between the high and low accessions. These transcripts matched those for a sucrose/ferredoxin-like protein, a putative RING-H2 finger protein associated with abscisic acid signalling, and a chloroplast-specific heat shock protein.

The comparison between high/low GLS accessions yielded five transcripts with at least one fixed site. These five loci corresponded to two beta glucosidases (*AT2G25630 & AT2G44450*), involved in carbohydrate metabolic processes; a protein kinase with potential function in salicylic acid biosynthesis (*AT5G47070*); a methyltransferase (*AT1G50000*); an MEK kinase (*AT1G53570*); and an unknown protein (*AT1G50020*).

## Discussion

Watercress is recognised as a crop with especially high concentrations of certain phytonutrients. These compounds not only confer the characteristic peppery taste associated with watercress, but are now considered to also offer important health benefits. However, limited knowledge exists on watercress genetics and genomics hindering efforts to preserve or select for these key traits. In this paper, we present the first transcriptome sequence for watercress that has utilised a unique germplasm resource collected globally and held currently at The University of Southampton [33]. We compiled a catalogue of over 80,000 watercress transcripts (57,349 annotated), described and compared the gene expression profile of ready-for-market watercress with contrasting antioxidant (AO) and glucosinolate (GLS) phenotypes and identified candidate genes for follow-up work, a subset of which may be useful in future watercress breeding. Some of the candidate genes identified in this analysis correspond to known metabolite pathways as well as others which require further investigation.

### Watercress transcriptome de novo assembly

Plants used in this study were harvested at the time point when the crop would be sent to market. The ten watercress samples with ‘extreme’ phytonutritional phenotypes and two control accessions were extracted for RNA and sequenced. NAS080, a commercial accession, was used to assemble a watercress reference transcriptome which comprised of 87,844 transcripts (trimmed to 80,800) and 48,732 corresponding “unigenes” (Table 3). For the *de novo* transcriptome assembly of the allohexaploid *Spartina* species, Ferreira de Carvalho et al. [46] applied a less stringent assembly in order to accommodate for up to six different alleles per sequenced locus. As watercress is thought to be tetraploid [47, 48], we also applied this approach. We conducted a variety of different assemblies which would potentially allow for the collapse, if present, of four alleles per locus into one. However, allowing for 0 to 8 differences (SNPs) within a path made no notable differences in transcripts or genes compiled among assemblies (Table 3). This would suggest that the watercress genome, if polyploidy, is likely to be autopolyploid, which would allow for duplicate polyploid genes (if expressed) to collapse into one regardless of assembly allowances.

By BLAST query against *Arabidopsis*, a close relative to watercress, coding regions we were able to annotate 70.6 % of the transcripts (57,075 of 80,800 transcripts) with an *Arabidopsis* locus identifier. Only 0.4 % of transcripts had a top hit in other plant species. For broccoli, another member of the Brassicaceae, 77.0 % of *de novo* assembled transcripts were annotated based on homology to *Arabidopsis* using an e-value of e^−5^ [49]. In our analysis, there were several cases were multiple watercress transcripts matched the same *Arabidopsis* locus identifier. This is likely to be a result of different fragments of the same transcript not being joined into a single transcript during assembly and/or gene duplication or loss in the lineage leading to one of the species. The transcripts that were not successfully annotated could be transcripts not shared with *Arabidopsis*, unique to watercress or incompletely assembled.

Watercress is assumed to be primarily self-fertilizing and spreads through clonal growth and root expansion. Commercial watercress is clonally propagated or selfed, since there is no current selection and breeding programme globally, so it is considered that watercress should have little genetic diversity. Thus, we would expect low polymorphism between accessions. Our results are consistent with this hypothesis, with 22 % of transcripts containing a polymorphic site. Nucleotide diversity was low across the entire data set (mean π = 0.78 and mean θ = 0.87 per Kb). For comparison, transcriptome nucleotide diversity θ in cultivated and wild carrot roots was 0.56 and 0.64 per Kb respectively [50]. The common bean transcriptome nucleotide diversity was greater than watercress and, in a comparison of Mesoamerican wild and cultivated beans, the wild variety (π = 2.11, θ = 2.08 per Kb) had higher diversity than its cultivated counterpart (π = 0.85, θ = 0.83 per Kb) [51].

### Gene expression and antioxidant capacity in watercress

The AO trait is desirable in crops cultivated for human consumption and is of particular interest in leafy salads, with the links between consumption of high AO leaves and their disease-preventing properties now becoming established. The phenylpropanoid pathway is an important and well-characterized pathway associated with the production of secondary plant metabolites and dietary AO compounds [21] and here, thirty six transcripts matched 21 of 24 phenylpropanoid pathway *Arabidopsis* sequences queried. Although considered at the gene sequence level and not taken through to translation, our findings suggest well-conserved gene sequences between *Arabidopsis* and watercress in the phenylpropanoid pathway and represent an immediately useful catalogue of important genes likely contributing to the AO crop trait.

We also completed DE analysis on five high and five low AO ‘extreme’ samples to describe the character of this trait at the whole-transcriptome level. DE analysis between high and low AO watercress returned 145 DE transcripts from 23 GO categories which were significantly associated with plant immunity, response to stimuli and stress. This direct link between plant defences and AO profile is not surprising, considering most compounds contributing to plant AO capacity are secondary plant metabolites associated with the very plant functions highlighted by the GO results. This link has been confirmed in field conditions. A multi-year field study on cauliflower showed annually variable phytochemical and AO contents which were linked to climate and rainfall [52], also confirming a significant environment component to this trait. In our laboratory, a significant difference between AO capacity (FRAP assay) of watercress grown in the field and in controlled environments has been identified, with field samples being overall higher [33]. These studies confirm that the synthesis and accumulation of secondary metabolites, underpinning the increase in AO capacity, is linked to plant response to external stimuli and stress (i.e. abiotic environmental stress or biotic stress through predation or pathogens). Thus, plant stress and immunity response genes and pathways should be considered strong candidates for breeding high AO food crops.

Although AO assays such as FRAP and ORAC provide a consistent measure of total AO capacity [53], the assays are unable to provide significant details on the specific compounds present that underpin AO capacity. This is a disadvantage when seeking a particular compound or pathway to attribute this health benefit but useful in overall characterisation of the consumer benefit derived from a crop. Thus, the phenotype we have assessed here represents a combination of multiple compounds with AO properties, and may include polyphenols (anthocyanins, flavonols, isoflavonoids, catechins, caffeoylquinic acid), carotenoids (lycopene, β-carotene, lutein), tocotrienols, tocopherols and ascorbic acid [15]. Indeed, several of the DE transcripts corresponded to elements of these AO compound biosynthetic pathways. For example, ferulate 5-hydroxylase (*AT4G36220*) is a phenylpropanoid pathway enzyme involved in lignin biosynthesis [21, 54], three transcripts (*AT5G41040*, *AT2G28630*, *AT2G28670*) are associated with suberin biosynthesis, a cell wall polymer containing phenolic components [55], a putative carotenoid hydrolase (*AT4G15110*), and tyrosine aminotransferase (*AT2G24850*) which is involved in tocopherol synthesis [56].

### Genes and pathways associated with GLS content of watercress

GLS are secondary plant metabolites utilized in plant defences against herbivory and have been the subject of many studies in the Brassicaceae. They contribute to the peppery flavour as well as the strong phytonutritional profile associated with watercress, thus the pathways and genes involved in the biosynthesis and processing of these compounds are an important research and breeding target for this crop. GLS biosynthesis is well-studied and the enzymes and genes involved in these steps are well-described in *Arabidopsis* and *Brassica rapa* for aliphatic and indolic GLS [28, 57]. Here, sequences of known GLS pathway genes in *Arabidopsis* were successfully identified in watercress. Wang et al. [58] used RNASeq to identify GLS biosynthesis genes in radish taproots as, similarly to watercress, these compounds contribute to the dietary and flavour profile of the crop. The authors identified sequences in radish that matched *Arabidopsis* and *B. rapa* GLS gene sequences and suggested that these genes are well-conserved in the Brassicaceae family [58]. Our findings support this hypothesis, as all GLS pathway gene sequences were also identified in watercress. In addition, we identified transcripts in watercress matching the *Arabidopsis* myrosinase coding sequence. This catalogue is immediately useful for further study of GLS biosynthesis in watercress, as well as in breeding, for hunting allelic variation in germplasm collections.

In addition, we compared whole transcriptome gene expression of three high and three low GLS watercress. A total of 94 transcripts were DE for this phenotype. Twenty four of these did not have a BLAST hit in *Arabidopsis*. Although the DE genes for this trait did not contain any GO categories with immediately obvious connection to GLS biosynthesis and regulation, there were several DE genes which were interesting on a gene-by-gene basis. Specifically, two DE transcripts belonged to the shikimate pathway (*c33663_g1_i2* –similar to shikimate kinases, *c37926_G1_i6* – dehydroquinate-shikimate dehydrogenase). The shikimate pathway leads to the synthesis of chorismate which is the precursor to phenylalanine, from which gluconasturtiin is derived (see Results). This direct link prompted a further investigation of the shikimate and phenylalanine biosynthetic pathways genes for which we used the known *Arabidopsis* sequences to mine for orthologs in watercress. These results are depicted in Figs. 4, 5 and 6 and show greater expression of 15 out of 17 genes in the high GLS watercress suggesting increased flux through this pathway in the high GLS plants. The potential connection between the shikimate pathway output and GLS levels in a plant provides a direct and appealing link for further investigation and would be of particular breeding interest, as phenylalanine also feeds into the AO phenylpropanoid pathway.

The GLS content of any plant tissue is under both genetic and environmental controls and depends on a variety of factors and conditions, including developmental stage [49, 59, 60], environmental conditions [52], and pest/herbivore exposure [60, 61]. For watercress, studies have shown GLS content variation in response to soil nitrogen and sulphur [43], selenium [45], as well as light and temperature [62]. In another study, 62 varieties of Chinese cabbage assessed were found to vary ca. 20-fold in GLS content, suggesting an effect of genotype on GLS production and accumulation [63]. Despite this, the variation in germplasm collection reported here, when all material was grown under identical environmental conditions, suggests there is potential for selective breeding for higher GLS. In fact, such a breeding endeavour has been undertaken successfully in broccoli, where an enriched GLS crop was produced through molecular breeding techniques and was shown to be associated with enhanced chemopreventive activity [64]. More recently, Beneforte broccoli has been released to market having 2.5–3 times higher GLS content than other broccoli varieties [65].

It is clear that the controls involved in the regulation GLS biosynthesis and accumulation in plants are complex and interdependent [66]. Several DE genes in this study could be linked to relevant regulatory pathways, such as stress and immune response, development and life stage, and ion or light response. Interestingly, 13 of DE 93 loci identified were linked with stress or immune response in plants, including genes associated with abscisic acid, jasmonic acid and salicylic acid signalling; an ethylene response transcription factor; a heat shock protein; glutathione-S-transferase, which is involved in cell detoxification; and a carotenoid biosynthesis enzyme.

As discussed previously, watercress GLS concentrations have been shown to respond to certain soil nutrients [43, 45, 67]. We identified two genes involved in cadmium ion response (*AT4G08790 & AT4G10320*) that were DE between the high and low GLS plants. Watercress GLS content has also been shown to respond to light [62] and our list of DE loci included a carotenoid biosynthesis enzyme (*AT4G25700*), carotenoids play a key role in photosynthesis and protects plant photosynthetic machinery from light damage [68, 69], and a phototropic-response protein (*AT3G44820*).

Finally, there were several DE elements for the GLS phenotype that were related to developmental processes. We resolved two MYB transcription factors; Circadian 1 (AT5G37260) and the circadian rhythm putative transcription factor LHY (*AT1G01060*). Certain MYB transcriptional factors have been suggested to act in GLS biosynthesis regulation [66, 70]. However, these transcription factors do not appear to fit previously suggested MYB links to GLS regulation, instead both are involved in circadian rhythms. An additional two transcription factors were DE here: *AT1G11950*, which contains a jumonji domain and is associated with flowering time, and a transcription factor of unknown function (*AT2G42780*). A pectin lyase-like protein was also differentially expressed (*AT1G19170*). Pectin lyases, which are cell wall components, are thought to act in fruit ripening and senescence amongst other plant developmental processes [71]. These findings are in support of previous field results showing differences in tissue GLS concentration over time and plant maturity [59, 60].

## Conclusions

In conclusion, we present the first fully annotated whole transcriptome sequencing of the highly nutritious leafy crop, watercress. Differential expression analysis of ‘extreme’ samples was used to detect genes potentially important to key nutritional traits and identified transcripts pertaining to the shikimate, phenylpropanoid and GLS biosynthetic pathways. The transcriptome of watercress offers a valuable resource for comparative study of the Brassicaceae which contains many crops, several of which have unique nutrient qualities which benefit humans. This work furthers our understanding of key genes and pathways associated with phytonutrient phenotypes in watercress and the genomic resources gathered will allow for the development of markers for marker assisted selection and further molecular studies on watercress, with aims to inform industry and research.

## Methods

### Plant material and phenotyping

Twenty five watercress accessions, from the University of Southampton germplasm collection, were grown side by side at a field site in Spetisbury (50°48’46.8”N, 2°08’47.9”W), Dorset U.K., under standard watercress commercial cultivation conditions, as described previously [33]. Specifically, watercress is traditionally grown in shallow gravel beds with flowing spring water. After seven weeks, the time at which the crop would typically be harvested for market, leaf and stem tissue was collected from all watercress accessions. Tissue was snap frozen in liquid nitrogen, ground and stored at -80 °C until further use. The antioxidant (AO) capacity of each sample was evaluated using an adapted Ferric Reducing Ability of Plasma (FRAP) protocol [72], described by Payne et al. [53]. Sap was extracted using a QiaShredder homogenizer tube (Qiagen, www.qiagen.com) and spun at 13,000 for 5 min at 4 °C. Samples were plated in 96-well plate alongside a serial dilution of iron sulphate heptahydrate. FRAP reagent mix, containing acetate buffer, TPTZ (2,4,6-tripyrid-s-triazine/hydrochloric acid) and ferric chloride hexahydrate, was added and the plate read immediately on a spectrophotometer (Anthos Labtec Instruments) at 620 nm. The FRAP assay utilizes the colour change which occurs during the reduction of ferric to ferrous ion to quantify the AO capacity of a sap sample [72].

Glucosinolates (GLS) were extracted from snap-frozen and ground tissue in 10 volumes of 70 % methanol at 70 ͦ C. Sinigrin, a GLS not found in watercress [73] was added as an internal standard at a concentration of 10 μg ml^−1^. Samples were incubated at 70 °C for 30 min with periodic mixing. The liquid phase was removed and centrifuged at 1 °C at 16,000 g for 5 min. Supernatants were transferred to amber vials and analysed by HPLC-MS. 10 μl of each extract was injected onto a Synergi Hydro-RP 150 × 2.0 mm column (Phenomenex, Macclesfield, UK) using an Accela autosampler (Thermo Fisher Scientific, UK). The mobile phase comprised 0.1 % (v/v) formic acid in water (solvent A) and 0.1 % formic acid in methanol (solvent B) pumped at 200 μl min^−1^ using an Accela 600 pump. The mobile gradient comprised an isocratic phase of 100 % solvent A for 4 min then a ramp to 20 % B over the next 10 min which was held for a further 6 min. A second ramp increased solvent B to 50 % over 5 min and was held for a further 10 min. Finally, solvent B was increased to 80 % over 5 min and held for a further 2 min prior to requilibration at 100 % before injection of the following sample. Column eluent was monitored using an Accela PDA and GLS were identified and quantified by ESI-MS and MS2 in negative ion mode using an LCQ fleet ion trap mass spectrometer according to Rochfort et al. [74]. The mass spectrometer was tuned against sinigrin using a sheath gas flow of 25, an auxiliary gas flow of five, a spray voltage of 5 kV and a capillary temperature 275 °C.

Phenotype data from the above procedures was used to categorize ‘extreme’ samples for differential expression analysis and is shown in Table 1. The five samples with the highest and lowest AO capacity were selected for sequencing. From these, the samples with the three highest and three lowest concentrations of gluconasturtiin were used for differential expression analysis. Two control accessions were also sequenced but not used in gene expression analysis. The first, NAS080, is a Vitacress Salads Ltd commercially-active accession that is widely sold across the U.K. and grown in the U.K., U.S.A., Portugal and Australia. The second control accession was NAS065, an accession from the University of Southampton germplasm collection which is of breeding interest because it exhibits the desirable phenotypes of high phytonutrient content and dwarf size.

### RNA extraction and Illumina sequencing

RNA was extracted with the RNeasy Mini kit (Qiagen, www.qiagen.com) and tested by nanodrop (Thermo Scientific ND-1000) and Agilent 2200 TapeStation (Agilent Technologies, www.agilent.com) for concentration, purity and integrity. Samples were sent to the Wellcome Trust Centre for Human Genetics, where they were converted to cDNA, A-tailed and adapter-ligated. The 12 samples were individually barcoded, combined and then pair-end sequenced in one lane of an Illumina Hiseq2500 (Illumina, www.illumina.com) producing 100 nt length reads. Initial quality checks were carried out using the standard Illumina pipeline.

### Processing and de novo assembly

Sample barcodes and poor quality reads (Q <15) were removed using cutadapt v1.5. NAS080 was chosen to assemble the reference transcriptome for watercress because it is an important commercial accession. We used *de novo* assembly software, Trinity, version 20140717 [36]. Data was *in silico* normalised to limit copies of each k-mer to 30, increasing the efficiency of the assembly by reducing run time and memory requirements [75]. The normalised reads were then assembled multiple times using various settings. As watercress is a tetraploid [47], four alleles could potentially be determined for each gene. Assemblies which permit greater numbers of differences per path could potentially collapse these paralogous genes or multiple alleles into one assembled component. We tested assemblies which allowed from 0 to 8 differences (SNPs) per path to expose such patterns in our data. Of these, we took forward the assembly with minimum k-mer coverage of two, maximum gap allowed per path of 15 bases, and two differences allowed per path (see Results).

The resulting assembly was then trimmed to filter out low count transcripts that are likely to be errors. The 12 sample libraries were mapped back to the reference transcriptome using RSEM in Trinity to determine FPKM (Fragments per kilobase of exon per million fragments mapped – a standardized value of expression) for each isoform of each gene. These data were then used to examine the effects of various trimming parameters. A trim with settings of minimum IsoPct (% expression of a transcript compared to other transcripts) of 1 % and a minimum FPKM of 1 was selected to be carried forward. The 12 individuals were then mapped back to the trimmed reference assembly, using RSEM, in order to examine gene expression variation between the individuals.

### Classification of transcripts and candidate gene identification

As watercress is closely related to *Arabidopsis* [76, 77]*,* the trimmed transcriptome was in first instance annotated using the *Arabidopsis* genome which has been fully sequenced and well-described [78, 79]. We used the software BioEdit Sequence Alignment Editor version 7.2.5 [80] to perform a BLASTx peptide search against current *Arabidopsis* peptide sequences, available from the TAIR database (file name: TAIR10_pep_20101214.fas). A cut-off e-value of e^−20^ was applied. Locus identifiers were then used to retrieve GO terms using the GO Annotation tool on the TAIR website (http://www.arabidopsis.org/tools/bulk/go/index.jsp). The transcriptome was further annotated using BLASTx search via Trinotate (http://trinotate.github.io/) against the UniProtKB/SWISS-PROT database [81] to annotate transcripts without a match and the UniProt ID mapping tool was used to retrieve gene identifiers and protein descriptions (http://www.uniprot.org/uploadlists/).

The mitochondrial and chloroplast genome of *Arabidopsis* was also compared to the transcripts in order to identify any transcripts that may have originated from non-nuclear DNA. Transcripts with a 95 % or higher sequence match and minimum 100 bases hit length were identified and not included in further analyses or interpretation.

A literature search was conducted to compile a list of genes involved in the phenylpropanoid pathway and in GLS biosynthesis, two major pathways directly linked with the traits of interest. For the GLS pathway, the gene list compiled in Wang et al. [57] for the watercress relatives *Arabidopsis* and *Brassica rapa* L. var. *silvestris* [Lam.] Briggs was used. In addition, we searched for matches to myrosinase (thioglucoside glucohydrolase), which is responsible for the conversion of GLS to the beneficial isothiocyanates upon consumption, in the annotated watercress transcriptome. The *Arabidopsis* gene sequences were retrieved from NCBI and then used in a BLASTn search of the watercress transcriptome. Orthology of best matches was further confirmed by a reciprocal BLAST of each watercress candidate sequence using the NCBI online BLAST tool (blast.ncbi.nlm.nih.gov/Blast.cgi).

### Differential expression analysis

Differential expression (DE) analysis was completed on standardized abundance estimates of transcripts for traits underpinning AO capacity and GLS content in Trinity, which utilises edgeR [41]. The five watercress samples with the highest and lowest AO capacity were used in DE analysis for the AO trait and the three highest and lowest GLS concentration were used for DE analysis for the GLS trait. After correction for false discovery due to multiple hypotheses testing, DE loci with FDR ≤ 0.05 are reported as significant in this study. Using AgriGO (0.05 significance with chi-squared test and Bonferroni correction), the GO terms of DE genes were compared to those of the reference transcriptome in order to identify over-represented GO categories. Fixation index (F_ST_) was also calculated between groups, using ProSeq3 [37], to guide the identification of potential polymorphisms associated with each trait.

### Genetic relatedness and polymorphic marker development

Following RSEM alignment of reads to the reference transcriptome,.bam files were exported. SAMtools [82] (settings: mpileup -q 3 -Q 20 -D –u), bcftools, vcfutils.pl (setting: -d 3) and seqtk were used to score polymorphisms relative to the reference transcriptome and create fasta files for polymorphism assessment. Polymorphisms were identified within ProSeq3. ProSeq3 was also used to calculate nucleotide diversity indices: π [38] and θ [39, 40]. In addition, the script misa.pl (http://pgrc.ipk-gatersleben.de/misa/) was applied to search for SSRs in the reference transcriptome, with the minimum repeat number of 8, 6, and 4 di-, tri- and tetranucleotides, respectively. A phylogenetic analysis of the accessions was completed using phyml (http://www.atgc-montpellier.fr/phyml/) and based on 7000 SNPs.

## Additional files

Additional file 1: Table S1. Complete annotation and differential expression data for watercress transcripts assembled and analysed in this study. (XLSX 12441 kb) Additional file 2: Figure S1. Genetic variation amongst the watercress accessions used in this study. (PDF 12 kb) Additional file 3: Table S2. Raw abundance estimates for each locus and sample in the antioxidant differential expression comparison. (XLSX 6056 kb) Additional file 4: Table S3. Raw abundance estimates for each locus and sample in the glucosinolate differential expression comparison. (XLSX 3990 kb)

## Abbreviations

AO: antioxidants
DE: differentially expressed
GLS: glucosinolates
NGS: next generation sequencing
