# Supplementary figures and images for: Characterization of the watercress (Nasturtium officinale R. Br.; Brassicaceae) transcriptome using RNASeq and identification of candidate genes for important phytonutrient traits linked to human health
Source: BMC Genomics. 2016 May 20;17:378. doi: 10.1186/s12864-016-2704-4 (PMC4875719; doi:10.1186/s12864-016-2704-4)

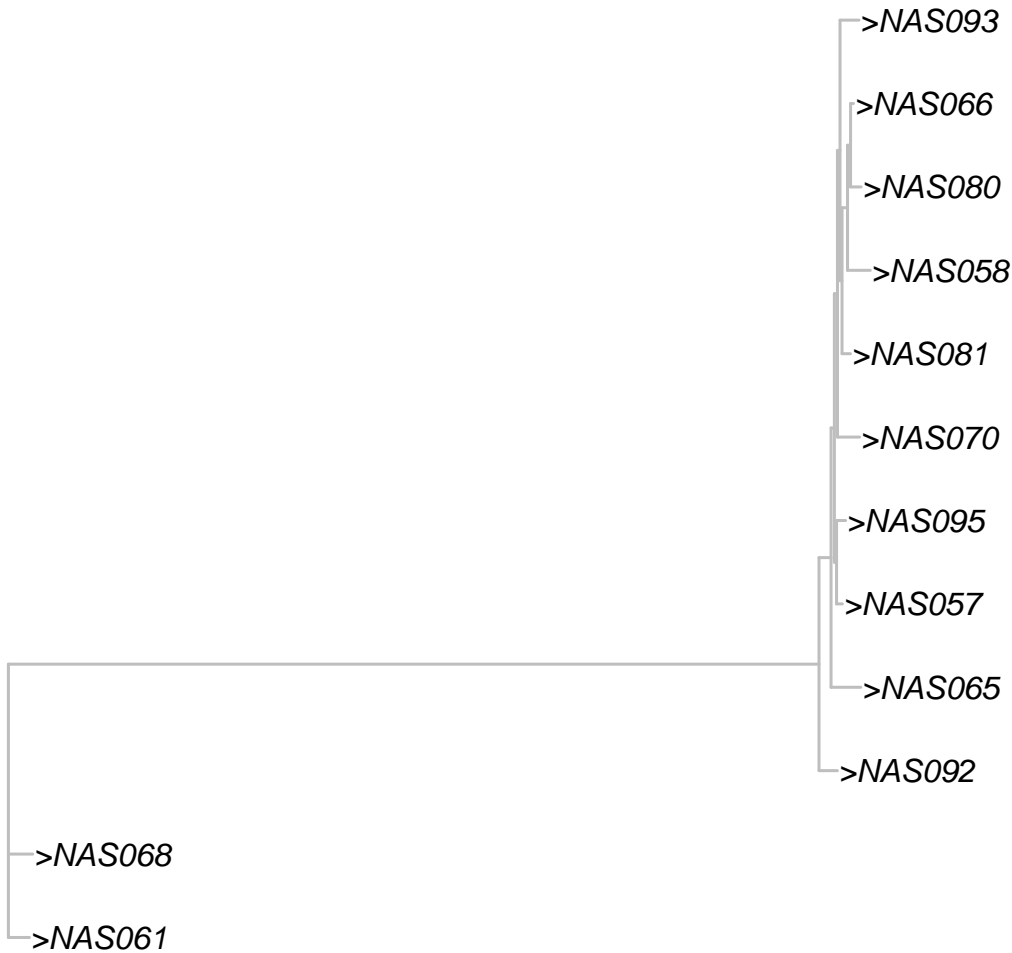

Supplement: Additional file 2: Figure S1. — Genetic variation amongst the watercress accessions used in this study. (PDF 12 kb) [file 12864_2016_2704_MOESM2_ESM.pdf]
